# BC-miR: Monitoring Breast Cancer-Related miRNA Profile in Blood Sera—A Prosperous Approach for Tumor Detection

**DOI:** 10.3390/cells11172721

**Published:** 2022-08-31

**Authors:** Barbara N. Borsos, Zoltán G. Páhi, Zsuzsanna Ujfaludi, Farkas Sükösd, Alíz Nikolényi, Sarolta Bankó, Gabriella Pankotai-Bodó, Orsolya Oláh-Németh, Tibor Pankotai

**Affiliations:** 1 Institute of Pathology, Albert Szent-Györgyi Medical School, University of Szeged, 6725 Szeged, Hungary; 2 Centre of Excellence for Interdisciplinary Research, Development and Innovation University of Szeged, 6723 Szeged, Hungary; 3 Genome Integrity and DNA Repair Group, Hungarian Centre of Excellence for Molecular Medicine (HCEMM), University of Szeged, 6728 Szeged, Hungary

**Keywords:** miRNA, biomarker, serum, breast cancer

## Abstract

Breast cancer is the most frequent cancer with a high fatality rate amongst women worldwide. Diagnosing at an early stage is challenging, and due to the limitations of the currently used techniques, including mammography and imaging diagnostics, it still remains unascertained. Serum biomarkers can be a solution for this as they can be isolated in a less painful, more cost-effective, and minimally invasive manner. In this study, we shed light on the relevant role of multiple microRNAs (miRNAs) as potential biomarkers in breast cancer diagnosis. We monitored the expressional changes of 15 pre-selected miRNAs in a large cohort, including 65 patients with breast cancer and 42 healthy individuals. We performed thorough statistical analyses on the cohort sample set and determined the diagnostic accuracy of individual and multiple miRNAs. Our study reveals a potential improvement in diagnostics by implicating the monitoring of miR-15a+miR-16+miR-221 expression in breast cancer management.

## 1. Introduction

Breast cancer is the most prevalent cancer worldwide. Based on the World Health Organization (WHO) data, in 2020, 2.3 million women were diagnosed with breast cancer, and 685,000 died of the disease. Early diagnosis is indispensable for increasing the survival rate and efficacy of the treatment. Nowadays, although tumor detection during mammography screenings may diminish the number of fatalities by 20%, in most cases, the recognition of a tumor in its early stage is challenging [1]. The most frequently used method for tumor confirmation is the biopsy, which is a painful and inconvenient procedure for the patient. Apart from tissue sampling, imaging diagnostics is also required, of which the resolution is often not enough to recognize the early-stage tumors; therefore, certain tumor types (e.g., lobular carcinoma) can mainly be detected in their late stages when they have already metastasized. Moreover, upon imaging screening, iodine-based or gadolinium contrast may have toxic side effects [2]. In breast cancer hormone therapy—if the patient is not suffering from triple-negative breast cancer (TNBC)—estrogen receptor (ER), progesterone receptor (PR), and human epidermal growth factor receptor 2 (HER2) are proven to be potential targets, and several biomarkers are successfully used for revealing their presence or absence in a given tumor [3]. Currently, there is no available early detection method that can be used for tumor prevention before the above-mentioned technologies may need to be applied. Serum biomarkers have the potential to recognize the tumor even in its very early stage—tumor extension is 10–15 mm without any metastases—when it can be treated with a high prevalence and without a possible recurrence [4,5]. This assumption has been supported by a recently published article about monitoring circulating tumor DNA (ctDNA) from plasma and highlighting the importance of this technology in pre-indicative breast cancer management [6].

microRNAs (miRNAs) are present in extracellular body fluids, including blood serum, blood plasma, saliva, and urine [7,8,9,10]. These circulating miRNAs are extremely stable due to their vesicle-associated subcellular location [11]. They can be found in exosomes, apoptotic bodies, or bound to Argonaute proteins [12]. Being stable, they can be easily detected by minimally invasive techniques, such as blood taking, then subsequent miRNA isolation and reverse-transcription quantitative real-time PCR (RT-qPCR) [4,5]. Hence, they are prospective biomarkers for tumor prognosis, early diagnosis, or potential therapeutic approaches.

According to the miRBase database, more than 2600 human miRNAs have been hitherto discovered [13]. They can post-transcriptionally regulate the expression of several genes, including cancer-related genes, through either mRNA degradation or translational blocking [2]. miRNAs are between 18 and 25 nucleotides long, small non-coding RNAs whose roles have been described in several processes, including physiological (e.g., development) and pathological conditions (e.g., neurodegenerative disorders). Apart from these, miRNAs have been demonstrated to play an important role in tumor progression [14]. It can be partially due to the fact that they are frequently encoded in the close proximity of cancer-associated genomic regions (such as common fragile sites), which may lead to chromosome disorders. According to their progression- or suppression-promoting function, miRNAs can act as either oncogenes (referred to as oncomiRs) or tumor suppressors, respectively [2]. Although our knowledge is continuously expanding about their role in various tumor types, their application as biomarkers in cancer prognosis, diagnosis, or therapy has yet to be elucidated.

Based on literature data, in this study, 15 miRNAs (miR-7, miR-15a, miR-16, miR-21, miR-125b, miR-135b, miR-136, miR-155, miR-181a, miR-200a, miR-200c, miR-210, miR-221, miR-519d, and miR-613) were selected to measure their expression in blood sera from healthy female volunteers (*n* = 42) and BC patients (*n* = 65) [15]. Regarding their previously characterized properties in tumor tissues, they can act either as tumor suppressors (miR-7, miR-15a, miR-16, miR-125b, miR-136, miR-200a, miR-200c, miR-519d, and miR-613) or oncogenes (miR-21, miR-135b, miR-155, miR-210, and miR-221) [16]. Intriguingly, miR-181a can act both as a tumor suppressor and an oncomiR, depending on its current target in the cells [17]. Furthermore, many of them, including miR-7, miR-15a, miR-16, miR-155, miR-181a, and miR-210, were found to be implicated in DNA repair circuits, supporting their regulatory role in cancer development [18,19,20,21].

Overall, the above-mentioned 15 miRNAs were selected based on the following criteria: (I) previously showed altered expression in breast cancer samples, (II) possess measurable expression in blood sera, (III) approximately half of them are supposed to be tumor suppressors and the other half are reported as oncogenes, (IV) some of them have been implicated in DNA repair pathways, (V) the number of the chosen miRNA population should be manageable and give us the chance to get enough miRNAs with altered expression in BC patients (Table 1). Moreover, we statistically analyzed and clustered those miRNAs which presumably have diagnostic relevancy. Based on these analyses, we hereby demonstrate the use of multiple circulating miRNAs, highlighting the most relevant combination of miR-15a+miR-16+miR-221, as potential combinatorial biomarkers in breast cancer management.

## 2. Materials and Methods

### 2.1. Cohort Characteristics

**Figure 1:**
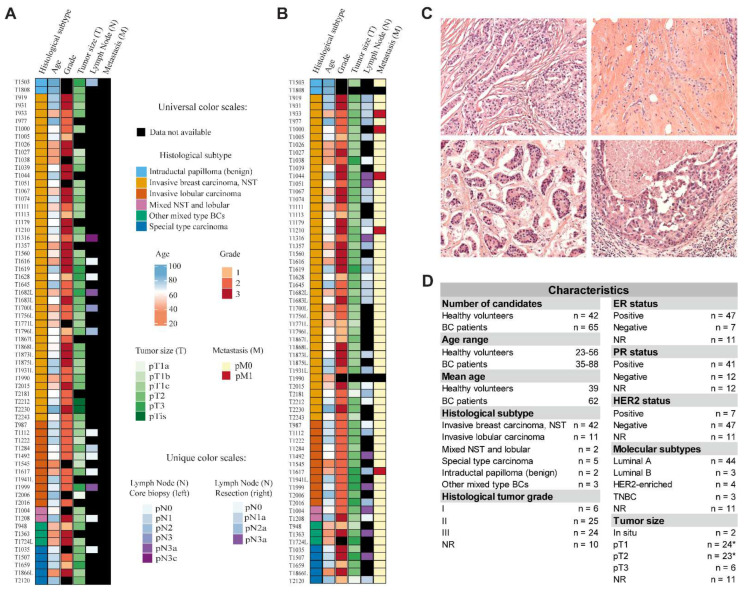
Physiological and pathological parameters of the individuals recruited for the study. (**A**) Heat map visualization of histological subtype, age, grade, tumor size (T), lymph node (N), and metastasis (M) obtained from BC patients from core biopsies. (**B**) Heat map visualization of histological subtype, age, grade, tumor size (T), lymph node (N), and metastasis (M) obtained from BC patients during surgical resection. (**C**) Hematoxylin and eosin staining of specimens belonging to invasive breast carcinoma (NST) (upper left), invasive lobular carcinoma (upper right), special type carcinoma (lower left), and other mixed type histological subtype (lower right). (**D**) Representation of physiological and pathological characteristics of the enrolled individuals. Asterisk indicates that a patient had both pT1 and pT2 tumors which are separately depicted in the table; NR = not recorded.

To begin, 42 healthy female volunteers with an average age of 39 (ranging from 23 to 56) and 65 BC patients with an average age of 63 (ranging from 35 to 88) were enrolled in our cohort study. Written informed consent was obtained from each participant. BC patients were classified according to their histological subtype (invasive ductal carcinoma, invasive lobular carcinoma, mixed ductal and lobular, special type carcinoma, intraductal papilloma, and other mixed type BCs), tumor grade (I–III), hormone receptor status (estrogen (ER), progesterone (PR), and human epidermal growth factor receptor 2 (HER2)), molecular subtypes (luminal A, luminal B, HER2-enriched, and triple-negative breast cancer (TNBC)), and tumor size (in situ, pT1–pT3). Of the patients, 66% were diagnosed with invasive ductal carcinoma, 17% of them with invasive lobular carcinoma, and the remaining patients were almost equally distributed among the other histological subtypes. Most of the patients were diagnosed with grade II and III tumors, indicating that the fast-growing appearance of breast cancer is more frequent. According to the hormone receptor status, most of the patients were positive for ER and PR, while negative for HER2 (Figure 1).

### 2.2. Hematoxylin and Eosin Staining of Tissue Specimens

Hematoxylin and eosin staining was performed based on the same protocol we published previously in Ördög et al. [37].

### 2.3. Preparation of Blood Sera

Blood sampling was carried out on BC patients, prior to the surgical intervention. For sera preparation, blood collection tubes with separation gel were used. Following blood taking, tubes were placed and incubated on ice from 30 to 60 min to preserve the integrity of miRNA molecules. Next, tubes were centrifuged at 2000 RPM for 10 min at 4 °C. The upper phase, which contained the blood serum, was carefully transferred (to avoid cross-contamination of the sera and the formed elements) and aliquoted to centrifuge tubes and stored at −80 °C. Hemolysis in each specimen was measured at 414 nm using a NanoDrop spectrophotometer. According to Michaela B. Kirschner et al., less than 0.2 cut-off values were determined to be non-hemolyzed [38]. If a sample had a ≥ 0.2 cut-off value, it was excluded from further analyses.

### 2.4. miRNA Isolation, Reverse Transcription, and qPCR

miRNAs were isolated from 200 µL of blood serum with the ReliaPrep™ miRNA Cell and Tissue Miniprep System (Promega) according to the manufacturer’s instruction. Then, a two-step reverse transcription (poly(A) tailing reaction and first-strand cDNA synthesis reaction) was assessed with MystiCq^®^ microRNA cDNA Synthesis Mix (Sigma-Aldrich, Darmstadt, Germany) based on the manufacturer’s protocol. As a reverse primer, MystiCq^®^ Universal PCR Primer (Sigma-Aldrich) was used for each qPCR reaction. Sequences of miRNAs were downloaded from the miRBase database. Forward primers were designed with miRprimer software [39] (Table S1). U6 primer was used as the endogenous control of the qPCR experiment; therefore, each relative expressional value was normalized to that of U6. GoTaq^®^ qPCR Master Mix (Promega, Madison, WI, USA) was used for qPCR detection. qPCRs were performed in duplicates.

### 2.5. Statistical Analyses

Expressional differences of miRNAs between BC and healthy individuals were assessed using the independent samples t-test (Table S2). Ascertaining the diagnostic accuracy of miRNAs, a receiver operating characteristic (ROC) curve was generated. The miRNA enrichment analysis and annotation tool (miEAA) 2.0 was used to assess the involvement of the desired miRNAs in breast cancer (Table S3). Box-plots were generated in SigmaPlot 12.5 software. Independent t-tests and ROC curve analyses were accomplished in IBM SPSS Statistics 28.0.

Determining the correlation between miRNAs, Pearson correlation coefficient (R) was conducted with stats package in R (version 4.1.1) using the ‘pairwise.complete.obs’ method for computing covariances [40]. The heatmaply R package with default settings was applied for visualizing the correlation results [41]. ColorRampPalette was used from the grDevices package by which colors were set with hexacodes [40].

The PCAtools R package (version 4.1.1) was used for the PCA analysis and PCA plots [42]. For the Principal Component Analysis, z-score normalized miRNA expressional data were used with the ‘scale=TRUE’ setting argument. For calculating the optimal number of clusters and creating PAM clustering on the z-score normalized miRNA expressional data, the factoextra R package was applied with default settings [43].

For calculating the accuracy, sensitivity, specificity, and prevalence values based on the confusion matrix in R, the confusion matrix argument was used from the caret package with default settings [44].

## 3. Results

### 3.1. Circulating miRNA Expression Level Changes between Patients with Breast Cancer and Healthy Volunteers

Expression level of 15 miRNAs (miR-7, miR-15a, miR-16, miR-21, miR-125b, miR-135b, miR-136, miR-155, miR-181a, miR-200a, miR-200c, miR-210, miR-221, miR-519d, and miR-613) was measured in blood sera from healthy female volunteers (*n* = 42) and BC patients (*n* = 65). Regarding the unequal distribution of patients within each pathological characteristic, we included all the BC patients in a single group and did not sort them into further subgroups.

**Figure 2:**
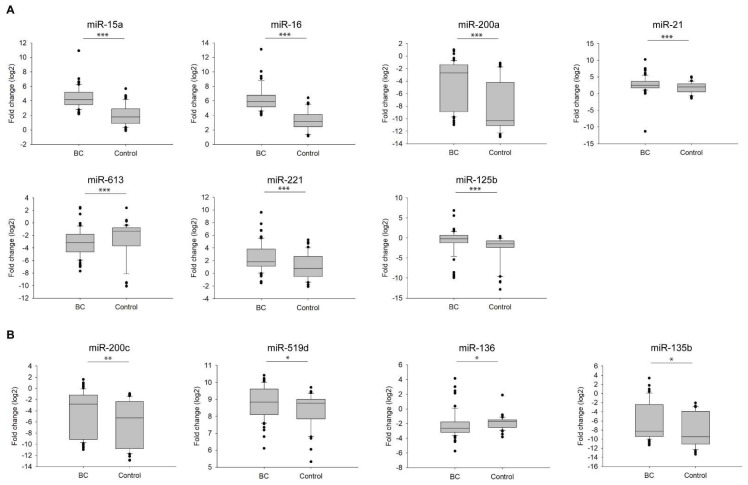
Fold change (log_2_) of miRNAs expression with significant alterations in BC patients (BC) compared with healthy individuals (Control). (**A**) Expressional analyses of miRNAs (miR-15a, miR-16, miR-200a, miR-21, miR-613, miR-221, and miR-125b) with a highly significant difference *** *p* < 0.001; (**B**) Expressional analyses of miRNAs (miR-200c, miR-519d, miR-136, and miR-135b) with a lower significant difference ** *p* < 0.01, and * *p* < 0.05. Error bars represent the standard deviation of each miRNA measured on either the group of BC patients (BC) or healthy individuals (Control).

To reveal which of the above-listed miRNAs appear to be promising biomarkers in breast cancer management, we isolated miRNAs from the blood sera of BC patients and healthy volunteers (previously verified that no hemolysis occurred), and then performed a reverse transcription-quantitative polymerase chain reaction (RT-qPCR). Differences between BC patients and healthy controls were represented in fold changes (log_2_) and statistically analyzed with independent *t*-tests. Results showed that, 7 miRNAs (miR-15a, miR-16, miR-200a, miR-21, miR-613, miR-221, and miR-125b) showed alterations with a high significance (*p* < 0.001) between BC patients and healthy candidates. Among them, 6 miRNAs (miR-15a, miR-16, miR-200a, miR-21, miR-221, and miR-125b) were upregulated, while miR-613 was downregulated in BC patients compared with healthy individuals (Figure 2A). These 7 significant miRNAs were further supported to be implicated in breast cancer by the miRNA enrichment analysis and annotation tool (miEAA) (Table S3) [45]; although we detected a less significant difference (*p* < 0.01 and *p* < 0.05) in the expression of the following miRNAs: miR-200c, miR-519d, miR-136, and miR-135b (Figure 2B). In the case of miR-155, miR-7, miR-181a, and miR-210 no significant difference was shown between BC patients and healthy volunteers (Figure S1A). Further studying the discrimination of the relevant miRNA candidates between BC patients and healthy individuals, we can establish a circulating miRNA profiling attributed to breast cancer.

### 3.2. Diagnostic Accuracy of Individual miRNAs

**Figure 3:**
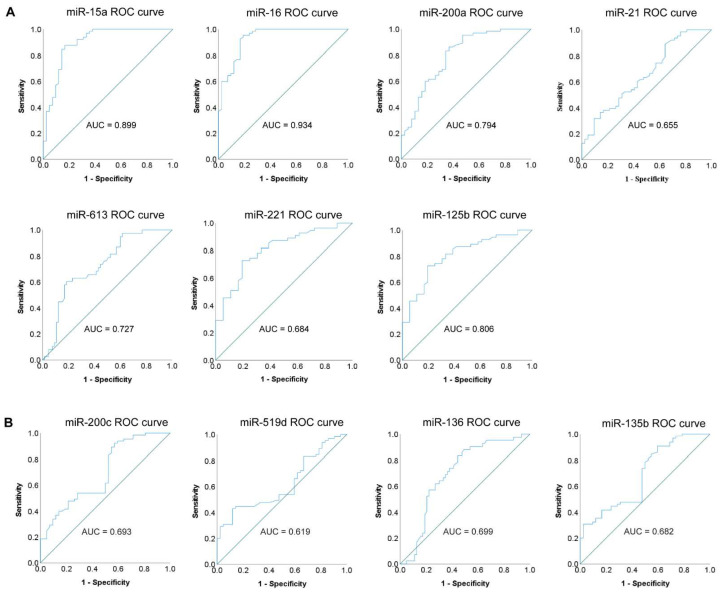
ROC curve analysis of individual miRNAs for validating their diagnostic accuracy. (**A**) ROC curves and the related AUC (area under the receiver operating characteristic curve) values of miRNAs (miR-15a, miR-16, miR-200a, miR-21, miR-613, miR-221, and miR-125b) with a highly significant difference; (**B**) ROC curves and the related AUC values of miRNAs (miR-200c, miR-519d, miR-136, and miR-135b) with a lower significant difference. Blue line represents the ROC curve, and the diagonal line depicts the line of no-discrimination.

ROC curve analysis was performed to determine the diagnostic accuracy of the miRNA candidates isolated from blood sera. The discriminatory power between BC patients and healthy volunteers was represented by the area under the receiver operating characteristic curve (AUC) (Figure 3and Figure S1B). miRNAs with AUC > 0.7 were accepted to possess potential diagnostic relevancies. Based on this restriction, miR-15a, miR-16, miR-200a, miR-613, and miR-125b seemed to have promising diagnostic potential (Figure 3A). Highlighting the most relevant miRNAs: miR-16 with an AUC of 0.934 and miR-15a with an AUC of 0.899 (Figure 3A). Although both have been described as tumor suppressor miRNAs, we found a significant increase in their level in BC patients compared with healthy controls (Figure 2A). As these miRNAs are important candidates for controlling cell proliferation, upon tumorigenesis, their tissue expression becomes lower (based on previously published data [46]), while their expression in sera increases, presumably due to their continuous secretion from the tissue into the blood where they cannot inhibit tumor growth. As assumed, those miRNAs which did not present significant differences between BC patients and healthy controls, neither had diagnostic potential (AUC < 0.65) (Figure S1B).

Based on the ROC curve analysis, we also determined the cut-off value, sensitivity, and specificity of the miRNA candidates. Emphasizing also in this case the two most relevant miRNAs with the highest specificity and sensitivity: miR-16 with 95.4% sensitivity and 81% specificity, and miR-15a with 87.7% sensitivity and 83.3% specificity (Table 2).

### 3.3. Correlation between the Desired miRNAs

**Figure 4:**
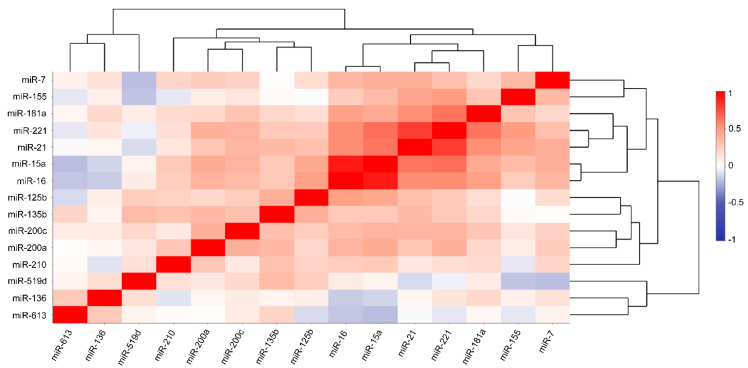
Pearson correlation coupled with agglomerative hierarchical cluster analysis (HCA) performed on all the desired miRNAs.

Unveiling the possible associations between the desired miRNAs, we used the Pearson correlation method illustrated as a heat map (Figure 4). The degree of correlation is determined based on the correlation coefficient (R): R > 0.7, strong correlation; 0.3 < R < 0.7, moderate correlation; R < 0.3, weak correlation. The most highly correlated miRNAs are miR-15a+miR-16 with R = 0.921 and miR-21+miR-221 with R = 0.805. We also verified the correlation between cross-groups (miR-15a+miR-21, miR-15a+miR-221, miR-16+miR-21, and miR-16+miR-221) of these four miRNAs. Although we found moderate correlation based on our strict ‘R-grouping’, these groups represented the highest correlations among others: (I) miR-15a+miR-21: 0.626, (II) miR-15a+miR-221: 0.647, (III) miR-16+miR-21: 0.548, and (IV) miR-16+miR-221: 0.548 (Figure 4 and Figure S2). R values of all possible miRNA correlation analyses and scatterplots (displaying the strength, direction, and form of relationship between the two variables) of miR-15a, miR-16, miR-21, and miR-221 are represented in Figure S3. Moreover, Pearson correlation and the related scatterplot analysis were conducted between miR-15a+miR-16 and miR-213miR-221 with R = 0.606 (Figure S3). Next, hierarchical cluster analysis (HCA), visualized by dendrograms, was assessed to sort the miRNAs into specific clusters and subclusters based on their correlations (Figure 4).

### 3.4. Expressional Changes and Diagnostic Accuracy of Multiple miRNAs

**Figure 5:**
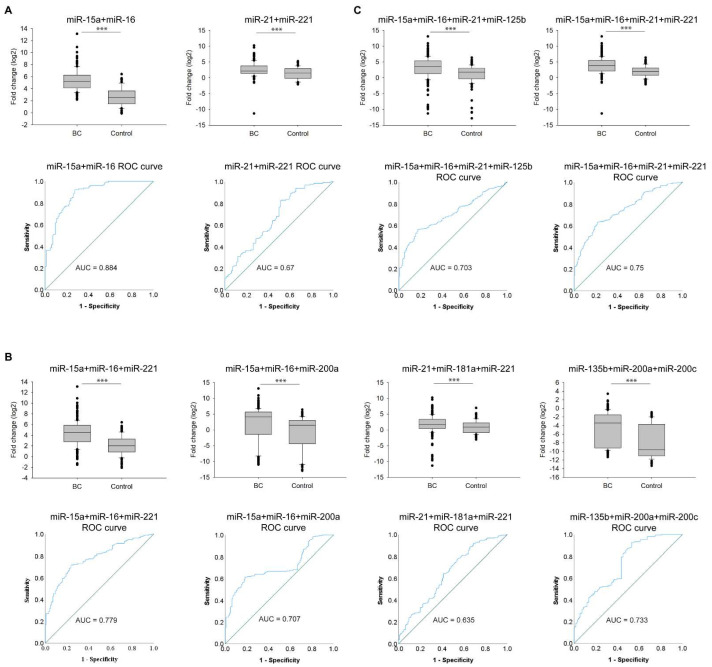
Fold change (log_2_) and ROC curve analysis of multiple miRNAs expression in BC patients (BC) compared with healthy individuals (Control). Combined expressional and ROC curve analyses of **A**) two correlated miRNAs, **B**) three correlated miRNAs, and **C**) four correlated miRNAs, *** *p* < 0.001. Error bars represent the standard deviation of each miRNA measured on either the group of BC patients (BC) or healthy individuals (Control). Blue line represents the ROC curve, and the diagonal line depicts the line of no-discrimination.

Based on the correlation analysis and HCA, we accomplished combined expressional ROC curve analyses on the cohort data to validate which combinations of the correlated miRNAs are supposed to be the most suitable for further diagnostics. We tested the combination of two miRNAs that presented the highest R values: miR-15a+miR-16 (AUC = 0.884) and miR-21+miR-221 (AUC = 0.67) (Figure 5A). Then, we investigated the results upon combining three correlated miRNAs: miR-15a+miR-16+miR-221 (AUC = 0.779), miR-15a+miR-16+miR-200a (AUC = 0.707), miR-21+miR-181a+miR-221 (AUC = 0.635), and miR-135b+miR-200a+miR-200c (AUC = 0.733) (Figure 5B). Finally, we examined the difference between BC patients and healthy individuals by the combination of four miRNAs: miR-15a+miR-16+miR-21+miR-125b (AUC = 0.703) and miR-15a+miR-16+miR-21+miR-221 (AUC = 0.75) (Figure 5C). Although miR-21+miR-221 showed the second strongest correlation value (Figure 4), based on the ROC curve analysis (AUC = 0.67), combining just the two of them did not seem to be a favorable biomarker combination. The most promising combinations of miRNAs are as follows: miR-15a+miR-16 with AUC = 0.884 (92.2% sensitivity and 72.6% specificity), miR-15a+miR-16+miR-221 with AUC = 0.779 (71.8% sensitivity and 76.2% specificity), and miR-15a+miR-16+miR-21+miR-221 with AUC = 0.75 (62.2% sensitivity and 78.6% specificity) (Figure 5 and Table 3). Apart from the valuable diagnostic accuracy, these combinations represented a normal distribution of the cohort data and a limited number of outliers, supporting their prosperous implication in breast cancer management. Albeit among these three combinations, miR-15a+miR-16 has the highest AUC value, in diagnostics, applying more than two biomarkers would have a greater therapeutic concern. Further supporting this, additional statistical analyses were conducted.

**Figure 6:**
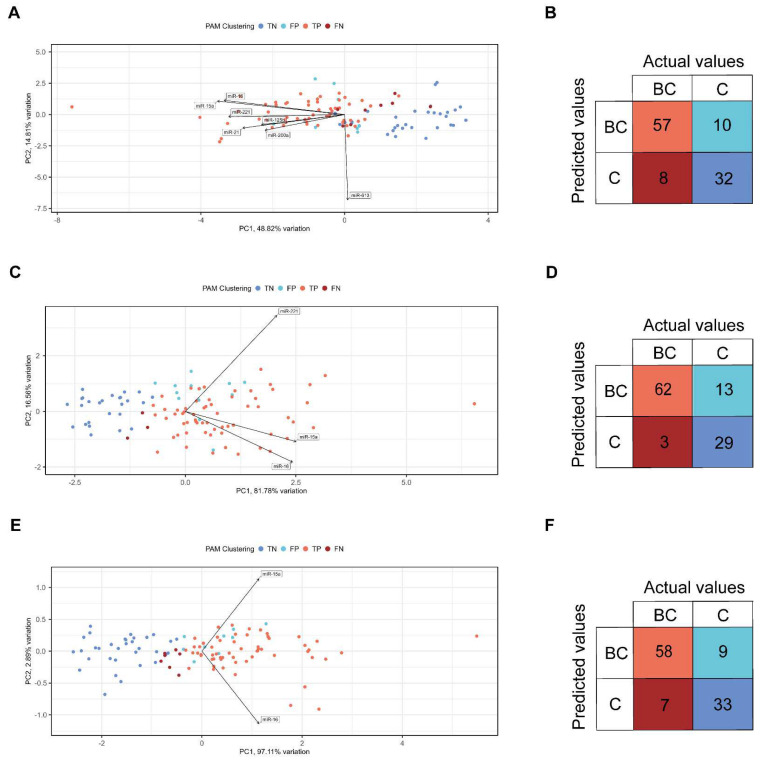
Principal component analysis (PCA) and confusion matrix of data derived from BC patients (BC) and healthy individuals (Control). (**A**), (**C**), and (**E**) PCA plots display the clustering data of (**A**) the 7 most significant miRNAs, (**C**) miR-15a+miR16+miR221, and (**E**) miR-15a+miR16, concerning the first two principal components (PC1 and PC2). The following eigenvalue clustering was applied: true positive (TP), true negative (TN), false positive (FP), and false negative (FN). (**B**,**D**,**F**), confusion matrixes represent the number of TP, TN, FP, and FN prediction values of (**B**) the 7 most significant miRNAs, (**D**) miR-15a+miR16+miR221, and (**F**) miR-15a+miR16. TP, TN, FP, and FN refer to the following outcomes, respectively: where the model (I) correctly predicts the BC patients; (II) correctly predicts the healthy individuals; (III) incorrectly predicts the BC patients when they are actually healthy individuals; and (IV) incorrectly predicts the healthy individuals when they are actually BC patients.

Principal component analysis (PCA) was performed to determine whether healthy individuals can be distinguished from BC patients based on the fold change values of the clustered miRNAs (all miRNAs (Figure S4A), the 7 significant miRNAs (Figure 6A), miR-15a+miR-16+miR-21+miR-221 (Figure S4C), miR-15a+miR-16+miR-221 (Figure 6C), and miR-15a+miR-16 (Figure 6E)). Eigenvalues (PCA scores of the samples displayed with colored dots) represent the specimens, and eigenvectors refer to the desired miRNAs in each PCA plot (Figure 6 and Figure S4). The summarized values of the first two principal components (PC1 and PC2) represent the total point variability as follows: (I) all miRNAs: 45.08% (Figure S4A); (II) 7 significant miRNAs: 63.63% (Figure 6A); (III) miR-15a+miR-16+miR-21+miR-221: 90.65% (Figure S4C); (IV) miR-15a+miR-16+miR-221: 98.34% (Figure 6C); and (V) miR-15a+miR-16: 100% (Figure 6E). By decreasing the number of miRNAs included in the certain clusters used to distinguish the BC and healthy categories, the total point variability is simultaneously getting increased. Those miRNAs whose related eigenvectors are the farthest from 0 mostly characterize the corresponding PC. Distance between the eigenvectors is consistent with the strength of correlation between the corresponding miRNAs (the less the distance between the eigenvectors, the stronger the correlation between the miRNAs). Eigenvectors point towards the eigenvalues corresponding to the PCA scores of BC patients, which support the potential involvement of these miRNAs in breast cancer. Based on the PCA analysis, BC and healthy clusters are well separated; therefore, we studied how these two categories can be distinguished with PAM clustering. The optimal cluster number turned out to be 2, determined by the Silhouette method (Figure S4E).

Further evaluating the performance metrics (accuracy, sensitivity, specificity, prevalence, etc.), based on PAM clustering, a confusion matrix (also known as error matrix) was constructed from the fold change values of all miRNAs (Figure S4B), the 7 significant miRNAs (Figure 6B), miR-15a+miR-16+miR-21+miR-221 (Figure S4D), miR-15a+miR-16+miR-221 (Figure 6D), and miR-15a+miR-16 (Figure 6F). Based on this matrix, we can determine the number of true positive (TP; outcome where the model correctly predicts the BC patients), true negative (TN; outcome where the model correctly predicts the healthy individuals), false positive (FP; outcome where the model incorrectly predicts the BC patients when they are actually healthy individuals), and false negative (FN; outcome where the model incorrectly predicts the healthy individuals when they are actually BC patients) prediction values which are as follows: (I) all miRNAs: 55 TP, 10 FP, 32 TN, and 10 FN (accuracy: 0.81, sensitivity: 0.85, specificity: 0.76, prevalence: 0.61) (Figure S4B); (II) the 7 most significant miRNAs: 57 TP, 8 FP, 32 TN, and 10 FN (accuracy: 0.83, sensitivity: 0.88, specificity: 0.76, prevalence: 0.61) (Figure 6B); (III) miR-15a+miR-16+miR-21+miR-221: 36 TP, 29 FP, 35 TN, and 7 FN (accuracy: 0.66, sensitivity: 0.55, specificity: 0.83, prevalence: 0.61) (Figure S4D); (IV) miR-15a+miR-16+miR-221: 62 TP, 3 FP, 29 TN, and 13 FN (accuracy: 0.85, sensitivity: 0.95, specificity: 0.69, prevalence: 0.61) (Figure 6D); (V) miR-15a+miR-16: 58 TP, 7 FP, 33 TN, and 9 FN (accuracy: 0.85, sensitivity: 0.89, specificity: 0.79, prevalence: 0.61) (Figure 6F).

According to the above-described analyses, miR-15a+miR-16+miR-221 multiple miRNAs turned out to be the most prominent biomarker combinations for further diagnostic validations in breast cancer management.

## 4. Discussion

In this study, we reveal a prosperous approach to breast cancer detection from blood sera. We emphasize the importance of recognizing breast cancer in the early stage, which may help increase the survival rate of BC patients. Being a minimally invasive technology, in contrast to the formally applied imaging technologies and biopsies, blood sampling is less painful and more cost-effective.

Mammography is the most commonly applied technology in breast cancer detection; however, it has many limitations, including providing either false positive or false negative results. Women with fatty or dense breasts have a higher chance of getting false results [47]. An advanced screening, digital breast tomosynthesis (DBT, also known as three-dimensional mammography), has been recently developed to resolve these constraints [48]; although DBT is not available in all breast imaging centers and requires a preliminary mammography screening, which is inconvenient and quite costly. Unlike mammography which uses X-rays for detection, magnetic resonance imaging (MRI) uses magnets and radio waves by which radiation exposure can be avoided and it can produce high-resolution images; nonetheless, its cost is even higher than that of the above-mentioned technologies [49]. An additional issue is that after being diagnosed with breast cancer, regular follow-ups are essential, which are accompanied by undergoing other rounds of inconvenient procedures that sometimes need to be repeated. A potential, but yet to be elucidated solution can be the involvement of miRNA profiling in personalized breast cancer management.

Being encapsulated in exosomes or bound with Argonaute proteins, circulating miRNAs are particularly stable; therefore, their implication for the development of novel biomarkers can be beneficial in targeted breast cancer therapy [12]. miRNAs are valuable candidates for establishing the diagnosis, monitoring the progression, and predicting the possible outcomes of the disease [50]. Moreover, they can even overcome drug resistance, indicating that they have the potential to get targeted during inhibitor targeted therapy, which may increase the therapeutic efficacy [51]. In our study, we revealed 7 significant individual miRNAs, and 5 of them are supposed to play a potential diagnostic role in breast cancer detection. Intriguingly, 6 miRNAs (miR-15a, miR-16, miR-200a, miR-21, miR-221, and miR-125b) were upregulated in BC patients regardless of their tumor suppressor or oncogenic role. The reason that the expressional changes of the desired miRNAs are not in concert with their tissue expression demonstrated by others could be that we measured the level of circulating miRNAs (and not their expression within the tissue) that had been secreted to the blood sera. These data support that the expression of miRNAs is regulated by different molecular mechanisms in tumor tissue and serum [46].

It is important to note that, generally, an individual miRNA can be characteristic of more than one tumor type; hence, to establish a breast cancer-specific miRNA profiling, the proper miRNA combinations should be determined. Accordingly, we generated different miRNA clusters, including various numbers of correlated miRNAs (*n* = 2–4), and monitored which ones seemed to be the most suitable for discriminating the healthy individuals from BC patients. To further validate the stratifying efficiency of the desired miRNA clusters, additional statistical analyses were conducted. These analyses underlined the promiscuous relevance of miR-15a+miR-16 and miR-15a+miR-16+miR-221 clusters in recognizing breast cancer with high prevalence and specificity. Although miR-15a+miR-16 demonstrated the highest specificity, these two candidates have already been shown to be involved in chronic lymphocytic leukemia [52]. Our aim was to reveal a miRNA cluster that tends to be specific to breast cancer; therefore, we included more than two strongly correlated miRNAs. Based on our statistical analyses, we determined the miR-15a+miR-16+miR-221 cluster as the most promising biomarker combination which is capable of discriminating the healthy volunteers from BC patients with a high probability. However, the miR-221 role is not restricted to breast cancer, its dysregulation has also been shown in hepatocellular carcinoma, pancreatic, and lung cancer [53,54,55]. In our cohort, the miR-15a+miR-16+miR-221 combination turned out to be the most promising multiple miRNAs, which may be suitable for further diagnostic purposes related to breast cancer. However, it is important to emphasize that in most cases, we examined the blood sera of BC patients with grade II and III, and unfortunately, regular follow-ups could not be performed due to the patients’ disastrously high and sudden mortality rate. In the future, the combination of these three biomarkers has to be further tested and monitored on a regular basis in a population possessing early-stage breast cancer. According to our data, we believe that if future validation studies supported our data, clinical implementation of miR-15a+miR-16+miR-221 would be of great importance in ameliorating the progression and recurrence of breast cancer.

## Figures and Tables

**Table 1:** Confirmed roles of the selected tumor suppressor and oncogene miRNAs in breast cancer. BC = breast cancer, Ref = Reference.

Potential Biomarker Candidates in Breast Cancer Management					
Tumor Suppressor miRNAs			OncomiRs		
miRNAs	Particular Role(s) in BC	Ref(s)	miRNAs	Particular Role(s) in BC	Ref(s)
**miR-7**	Suppression of mobility and invasiveness. Regulation of DNA repair.	[22,23]	**miR-21**	Promoting cell invasion.	[24]
**miR-15a**	P53-mediated expression. Involved in DNA repair.	[21,25]	**miR-135b**	Cell growth stimulation and cell cycle disruption.	[26]
**miR-16**	Cell cycle arrest and regulation of DNA repair.	[21,27]	**miR-155**	Promoting cell migration and invasiveness. Regulation of DNA repair.	[18]
**miR-125b**	Prevention of HER-2 overexpression. Suppression of cell proliferation.	[28,29]	**miR-181a**	Suppression of DNA damage response.	[19]
**miR-136**	Suppression of cell migration and invasion.	[30]	**miR-210**	Increasing cell invasiveness and DNA repair regulation.	[31]
**miR-181a**	Prevention of cancer metastasis.	[32]	**miR-221**	Promoting cell migration and invasion.	[33]
**miR-200a**	EMT inhibition.	[34]			
**miR-200c**	EMT inhibition.	[34]			
**miR-519d**	Suppression of cancer metastasis by targeting *MMP-3*.	[35]			
**miR-613**	Suppression of cell migration and invasion by targeting *Daam1*.	[36]			

**Table 2:** Representation of AUC values, cut-off values, sensitivity, specificity, standard error, asymptotic significance, and asymptotic 95% confidence interval (CI) of individual miRNAs based on the ROC curve analyses displayed in Figure 3.

	AUC	Cut-Off Value	Sensitivity	Specificity	St. Error ^a^	Asymptotic Sign. ^b^	Asymptotic 95% CI	
							Lower Bound	Upper Bound
miR-16	0.934	4.270	95.40%	81.00%	0.025	0.000	0.885	0.982
miR-15a	0.899	3.055	87.70%	83.30%	0.033	0.000	0.833	0.965
miR-125b	0.806	−0.670	72.70%	80.60%	0.046	0.000	0.717	0.896
miR-200a	0.794	−9.405	84.60%	63.20%	0.047	0.000	0.702	0.887
miR-613	0.727	−1.49	60.50%	81.50%	0.050	0.000	0.629	0.825
miR-136	0.699	−2.665	88.10%	49.20%	0.052	0.001	0.597	0.800
miR-200c	0.693	−9.94	92.30%	42.90%	0.053	0.001	0.589	0.796
miR-221	0.684	1.210	75.40%	61.00%	0.055	0.001	0.576	0.792
miR-135b	0.682	−9.940	90.80%	40.50%	0.053	0.001	0.578	0.787
miR-21	0.655	1.240	88.90%	35.70%	0.054	0.007	0.548	0.761
miR-7	0.64	−9.590	87.00%	50.00%	0.063	0.021	0.517	0.762
miR-519d	0.619	9.190	41.15%	88.10%	0.054	0.039	0.512	0.725
miR-210	0.614	−9.970	94.90%	35.00%	0.060	0.055	0.496	0.732
miR-181a	0.602	−0.855	81.70%	48.80%	0.060	0.083	0.485	0.719
miR-155	0.591	−9.580	88.70%	38.50%	0.061	0.127	0.471	0.710

^a^ Under the nonparametric assumption; ^b^ Null hypothesis: true area = 0.5.

**Table 3:** Representation of AUC values, cut-off values, sensitivity, specificity, standard error, asymptotic significance, and asymptotic 95% confidence interval (CI) of multiple miRNAs based on the ROC curve analyses displayed in Figure 5.

	AUC	Cut-Off Value	Sensitivity	Specificity	St. Error ^a^	Asymptotic Sign. ^b^	Asymptotic 95% CI	
							Lower Bound	Upper Bound
miR-15a+miR-16	0.884	3.305	92.20%	72.60%	0.024	0.000	0.838	0.930
miR-16+miR-15a+miR-221	0.779	2.323	71.80%	76.20%	0.025	0.000	0.729	0.829
miR-16+miR-15a+miR-21+miR-221	0.750	3.355	62.20%	78.60%	0.023	0.000	0.704	0.795
miR-135b+miR-200a+miR-200c	0.733	−9.975	92.80%	46.80%	0.029	0.000	0.677	0.79
miR-15a+miR-16+miR-200a	0.707	3.305	61.50%	81.70%	0.029	0.000	0.651	0.763
miR-15a+miR-16+miR-21+miR-125b	0.703	3.265	56.50%	81.40%	0.025	0.000	0.654	0.751
miR-21+miR-221	0.670	1.220	82.20%	48.80%	0.038	0.000	0.595	0.745
miR-21+miR-181a+miR-221	0.635	1.220	63.20%	58.70%	0.032	0.000	0.572	0.698

^a^ Under the nonparametric assumption; ^b^ Null hypothesis: true area = 0.5.

## Data Availability

Not applicable.

## Supplementary Materials

The following supporting information can be downloaded at: https://www.mdpi.com/article/10.3390/cells11172721/s1, Figure S1: Fold change (log_2_) and ROC curve analysis of non-significant individual miRNAs expression in BC patients (BC) compared with healthy individuals (control) Figure S2: Pearson correlation analysis performed on all the desired miRNAs; Figure S3: Pearson correlation analysis and the related scatter plot performed on miR-15a+miR-16 and miR-21+miR-221; Figure S4: Principal component analysis (PCA) and confusion matrix of data derived from BC patients and healthy individuals; Table S1: Primer sequences of miRNAs used for qPCR reactions; Table S2: n-, p-, and t-values as well as degrees of freedom (df) of independent t-tests executed on individual or multiple miRNAs; Table S3: miRNAs shown to be involved in breast cancer based on the miRNA enrichment analysis and annotation tool (miEAA).
