# Supplementary material for: BC-miR: Monitoring Breast Cancer-Related miRNA Profile in Blood Sera—A Prosperous Approach for Tumor Detection
Source: Cells. 2022 Aug 31;11(17):2721. doi: 10.3390/cells11172721 (PMC9454447; doi:10.3390/cells11172721)
Supplement: Supplementary file 1 [file cells-11-02721-s001.zip › cells-1835576-supplementary.pdf]

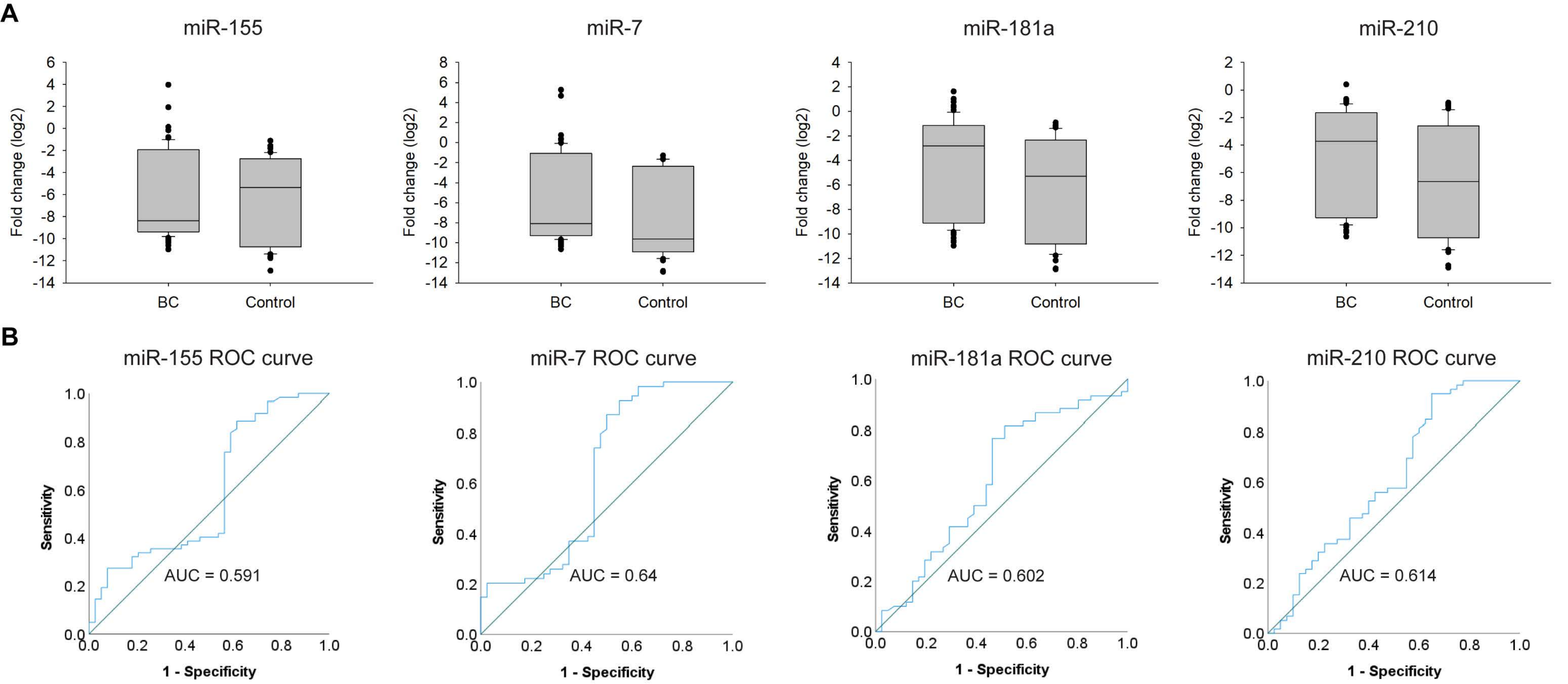

Figure S1 Fold change (log2) and ROC curve analysis of non-significant individual miRNAs expression in BC patients (BC) compared with healthy individuals (Control). A) Expressional analyses of non-significant miRNAs (miR-7, miR-155, miR-181a, and miR-210); **B)** ROC curves, and the related AUC (area under the receiver-operating characteristic curve) values of non-significant miRNAs (miR-7, miR-155, miR-181a, and miR-210). Error bars represent the standard deviation of each miRNA measured on either the group of BC patients (BC) or healthy individuals (Control).

Correlations

		miR125b	miR613	miR21	miR16	miR15a	miR136	miR519d	miR135b	miR200c	miR181a	miR221	miR200a	miR210	miR155	miR7
miR125b	Pearson Correlation	1	-.111	.319**	.476**	.437**	.092	.259*	.406**	.256*	.164	.268*	.197	.227*	-.001	.182
	Sig. (2-tailed)		.308	.002	<.001	<.001	.391	.013	<.001	.014	.132	.010	.065	.039	.989	.108
	N	91	87	91	91	91	89	91	90	91	86	91	88	83	85	79
miR613	Pearson Correlation	-.111	1	-.017	-.172	-.199*	.272**	.061	.215*	.107	.048	-.070	.010	.028	-.071	.065
	Sig. (2-tailed)	.308		.864	.082	.044	.006	.540	.030	.284	.639	.484	.924	.791	.487	.543
	N	87	103	101	103	103	100	103	102	103	97	102	99	95	97	90
miR21	Pearson Correlation	.319**	-.017	1	.548**	.626**	.052	-.112	.346**	.371**	.543**	.805**	.289**	.122	.452**	.405**
	Sig. (2-tailed)	.002	.864		<.001	<.001	.602	.254	<.001	<.001	<.001	<.001	.003	.234	<.001	<.001
	N	91	101	105	105	105	102	105	104	105	100	104	101	97	99	92
miR16	Pearson Correlation	.476**	-.172	.548**	1	.921**	-.155	.100	.279**	.345**	.474**	.548**	.387**	.257*	.257**	.369**
	Sig. (2-tailed)	<.001	.082	<.001		<.001	.116	.303	.004	<.001	<.001	<.001	<.001	.010	.009	<.001
	N	91	103	105	107	107	104	107	106	107	101	106	103	99	101	94
miR15a	Pearson Correlation	.437**	-.199*	.626**	.921**	1	-.130	.059	.276**	.383**	.430**	.647**	.423**	.275**	.341**	.404**
	Sig. (2-tailed)	<.001	.044	<.001	<.001		.189	.549	.004	<.001	<.001	<.001	<.001	.006	<.001	<.001
	N	91	103	105	107	107	104	107	106	107	101	106	103	99	101	94
miR136	Pearson Correlation	.092	.272**	.052	-.155	-.130	1	.168	.059	.105	.197	.135	.050	-.083	.088	.158
	Sig. (2-tailed)	.391	.006	.602	.116	.189		.088	.553	.288	.051	.173	.624	.422	.384	.133
	N	89	100	102	104	104	104	104	103	104	98	103	100	96	99	92
miR519d	Pearson Correlation	.259*	.061	-.112	.100	.059	.168	1	.345**	.199*	.103	-.047	.123	.160	-.177	-.194
	Sig. (2-tailed)	.013	.540	.254	.303	.549	.088		<.001	.040	.304	.631	.217	.114	.076	.061
	N	91	103	105	107	107	104	107	106	107	101	106	103	99	101	94
miR135b	Pearson Correlation	.406**	.215*	.346**	.279**	.276**	.059	.345**	1	.317**	.206*	.273**	.352**	.311**	.024	.014
	Sig. (2-tailed)	<.001	.030	<.001	.004	.004	.553	<.001		<.001	.039	.005	<.001	.002	.810	.898
	N	90	102	104	106	106	103	106	106	106	100	105	102	98	100	93
miR200c	Pearson Correlation	.256*	.107	.371**	.345**	.383**	.105	.199*	.317**	1	.296**	.382**	.399**	.115	.132	.234*
	Sig. (2-tailed)	.014	.284	<.001	<.001	<.001	.288	.040	<.001		.003	<.001	<.001	.258	.188	.023
	N	91	103	105	107	107	104	107	106	107	101	106	103	99	101	94
miR181a	Pearson Correlation	.164	.048	.543**	.474**	.430**	.197	.103	.206*	.296**	1	.630**	.199	.189	.297**	.197
	Sig. (2-tailed)	.132	.639	<.001	<.001	<.001	.051	.304	.039	.003		<.001	.051	.068	.004	.066
	N	86	97	100	101	101	98	101	100	101	101	100	97	94	95	88
miR221	Pearson Correlation	.268*	-.070	.805**	.548**	.647**	.135	-.047	.273**	.382**	.630**	1	.399**	.163	.494**	.323**
	Sig. (2-tailed)	.010	.484	<.001	<.001	<.001	.173	.631	.005	<.001	<.001		<.001	.108	<.001	.002
	N	91	102	104	106	106	103	106	105	106	100	106	102	98	100	93
miR200a	Pearson Correlation	.197	.010	.289**	.387**	.423**	.050	.123	.352**	.399**	.199	.399**	1	.287**	.091	.264*
	Sig. (2-tailed)	.065	.924	.003	<.001	<.001	.624	.217	<.001	<.001	.051	<.001		.004	.374	.011
	N	88	99	101	103	103	100	103	102	103	97	102	103	97	98	92
miR210	Pearson Correlation	.227*	.028	.122	.257*	.275**	-.083	.160	.311**	.115	.189	.163	.287**	1	-.070	.211*
	Sig. (2-tailed)	.039	.791	.234	.010	.006	.422	.114	.002	.258	.068	.108	.004		.497	.049
	N	83	95	97	99	99	96	99	98	99	94	98	97	99	95	88
miR155	Pearson Correlation	-.001	-.071	.452**	.257**	.341**	.088	-.177	.024	.132	.297**	.494**	.091	-.070	1	.349**
	Sig. (2-tailed)	.989	.487	<.001	.009	<.001	.384	.076	.810	.188	.004	<.001	.374	.497		<.001
	N	85	97	99	101	101	99	101	100	101	95	100	98	95	101	90
miR7	Pearson Correlation	.182	.065	.405**	.369**	.404**	.158	-.194	.014	.234*	.197	.323**	.264*	.211*	.349**	1
	Sig. (2-tailed)	.108	.543	<.001	<.001	<.001	.133	.061	.898	.023	.066	.002	.011	.049	<.001	
	N	79	90	92	94	94	92	94	93	94	88	93	92	88	90	94

**. Correlation is significant at the 0.01 level (2-tailed).

*. Correlation is significant at the 0.05 level (2-tailed).

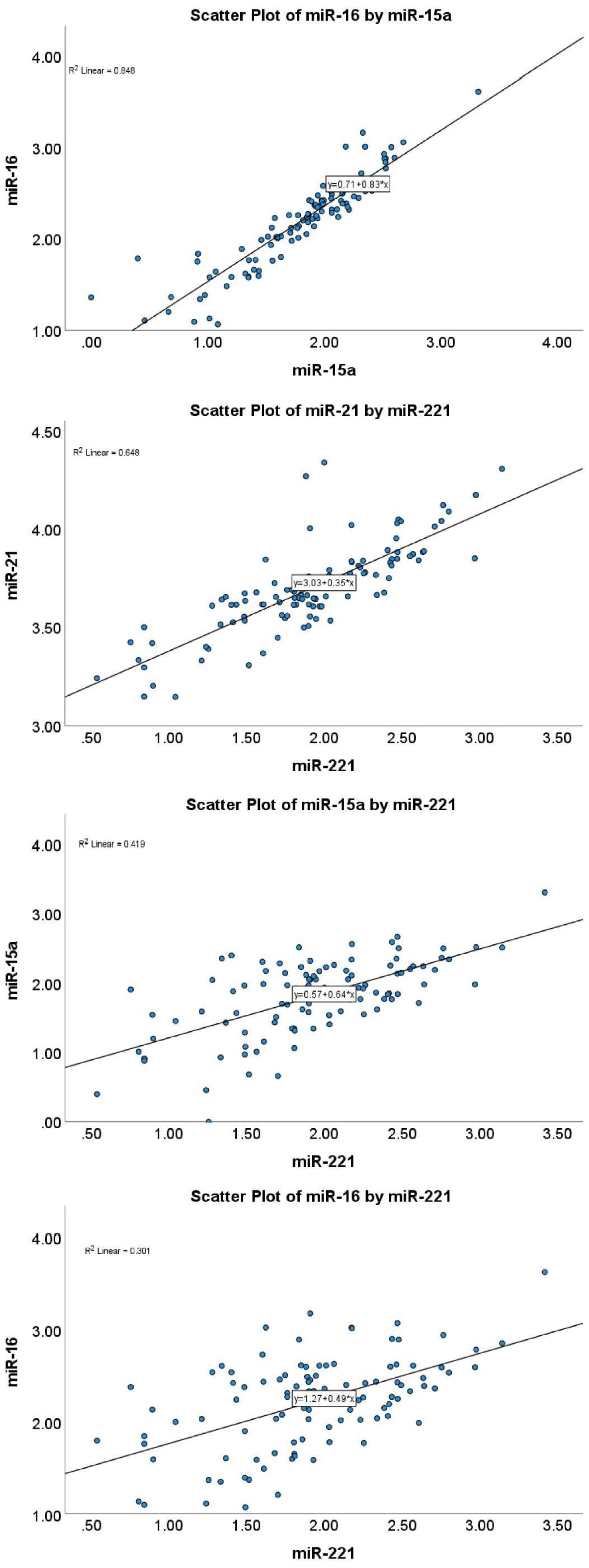

Figure S2 Pearson correlation analysis performed on all the desired miRNAs. Pearson correlation coefficient, p- and n-values are depicted in the correlation table. Scatter plots display the mostly correlated miRNAs, including miR-15a–miR-16, miR-21–miR-221, miR-15a–miR-221, and miR-16–miR-221.

Correlations

		miR21_miR221	miR16_miR15a
miR21_miR221	Pearson Correlation	1	.606**
	Sig. (2-tailed)		<.001
	N	213	212
miR16_miR15a	Pearson Correlation	.606**	1
	Sig. (2-tailed)	<.001	
	N	212	213

**. Correlation is significant at the 0.01 level (2-tailed).

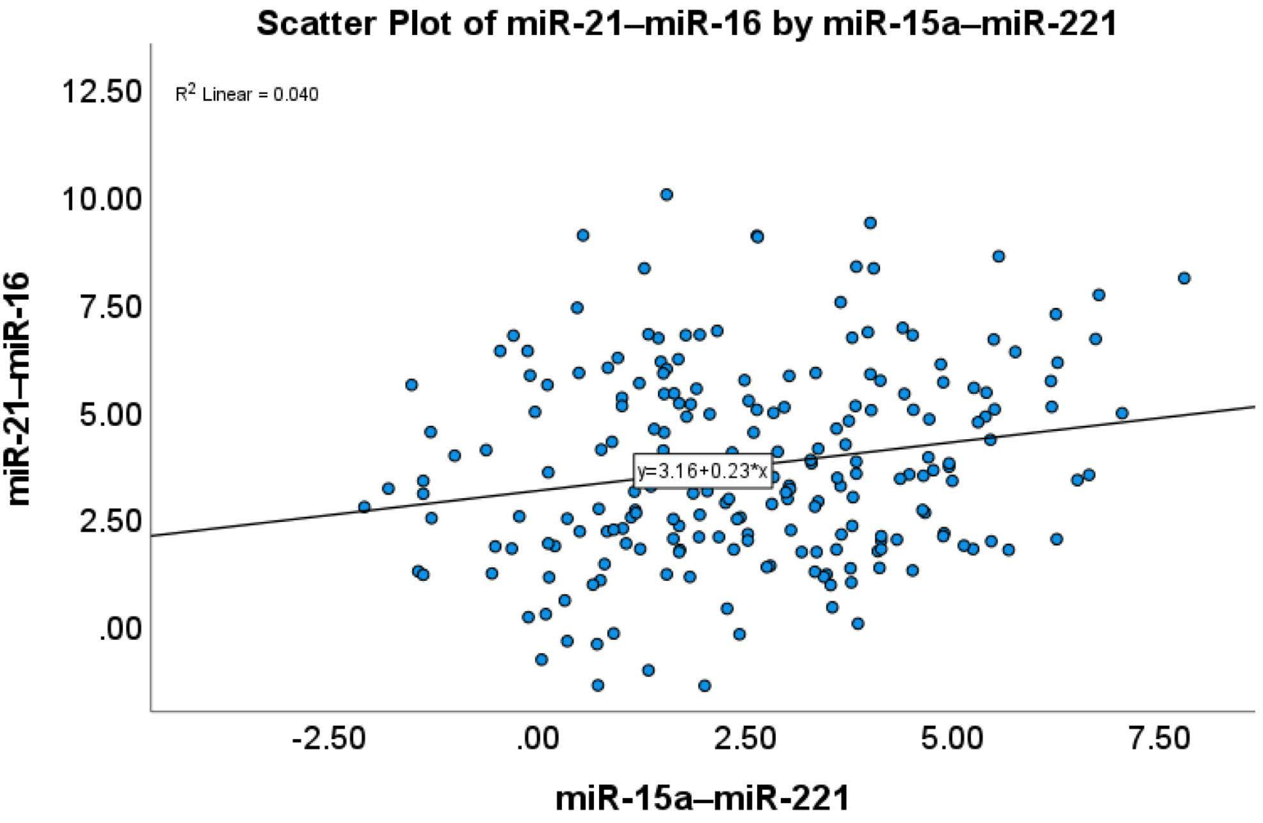

Figure S3 Pearson correlation analysis and the related scatter plot performed on miR-15a–miR-16 and miR-21–miR-221. Pearson correlation coefficient, p- and n-values are depicted in the correlation table.

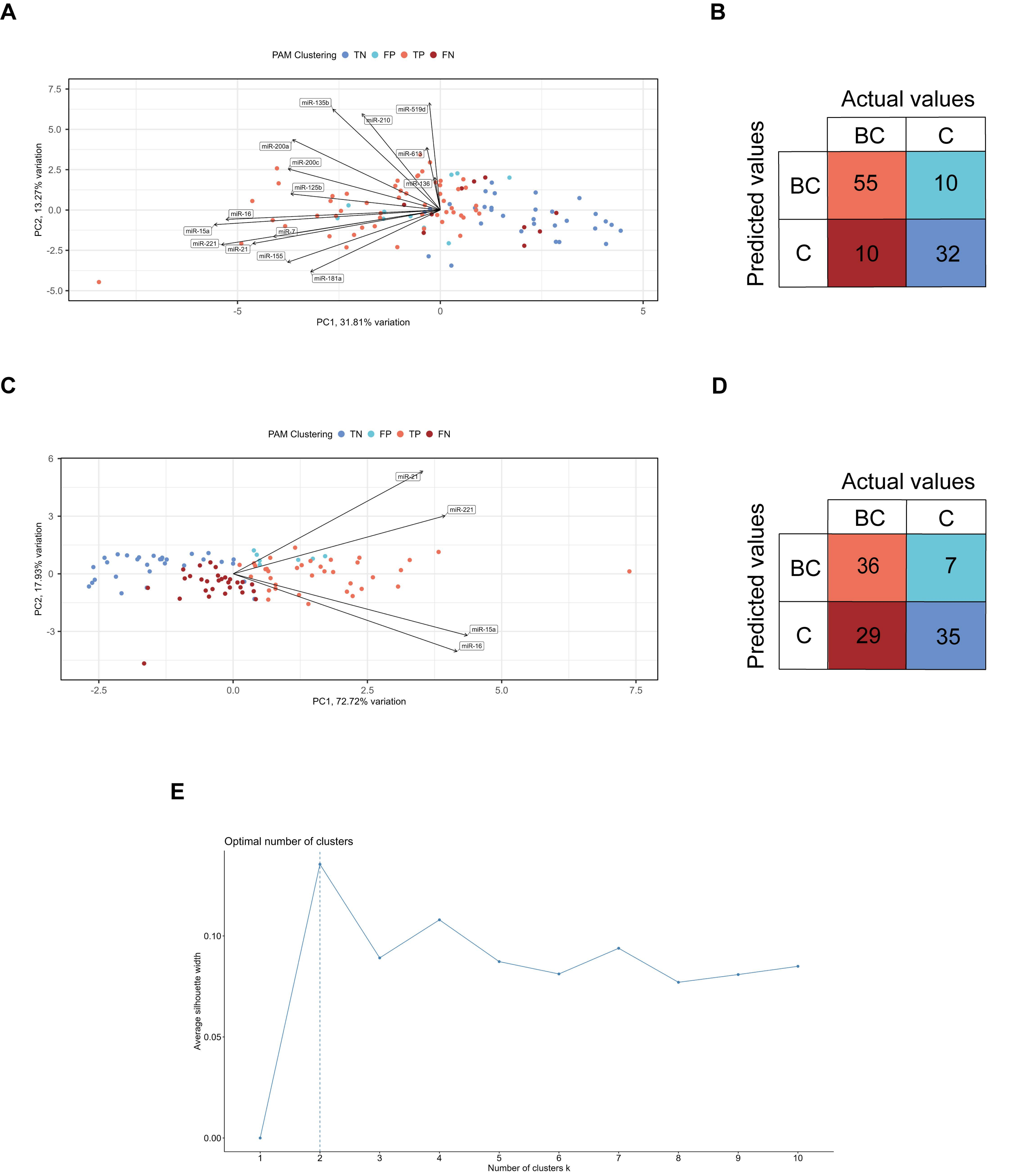

Figure S4 Principal component analysis (PCA) and confusion matrix of data derived from BC patients and healthy individuals. A) and **C)** PCA plots display the clustering data of (**A**) all miRNAs and (**C**) miR-15a+miR-16+miR-21+miR-221, concerning the first two principal components (PC1 and PC2). The following eigenvalue clustering was applied: true positive (TP), true negative (TN), false positive (FP), and false negative (FN). **B)** and **D)** Confusion matrixes represent the number of TP, TN, FP, and FN prediction values of (**B**) all miRNAs and (**D**) miR-15a+miR-16+miR-21+miR-221. TP, TN, FP, and FN refer to the following outcomes, respectively: where the model (I) correctly predicts the BC patients; (II) correctly predicts the healthy individuals; (III) incorrectly predicts the BC patients when they are actually healthy individuals; and (IV) incorrectly predicts the healthy individuals when they are actually BC patients. **E)** displays the optimal number of clusters determined by the Silhouette method.

	Fwd primer 5'-3'
miR-125b	GCAGTCCCTGAGACCCT
miR-7	TGGAAGACTAGTGATTTTGTTG
miR-15a	CAGTAGCAGCACATAATGGT
miR-16	TAGCAGCACGTAAATATTGG
miR-21	TAGCTTATCAGACTGATGTTGA
miR-200c	AGTAATACTGCCGGGTAATGA
miR-210	GCTGTGCGTGTGACA
miR-155	CGCAGTTAATGCTAATCGTGATAG
miR-181a	CATTCAACGCTGTCGGT
miR-613	GGAATGTTCCTTCTTTGCC
miR-519d	AGCAAAGTGCCTCCCT
miR-221	AGCTACATTGTCTGCTGGG
miR-200a	CAGTAACACTGTCTGGTAACG
miR-135b	GCAGTATGGCTTTTCATTCCT
miR-136	CGCAGCATCATCGTCTCA
U6	CTCGCTTCGGCAGCACATA

Table S1 Primer sequences of miRNAs used for qPCR reactions

	BC (n)	C (n)	p-value	t-value	df
**miR-15a**	65	42	<0.001	9.32	105
**miR-16**	65	42	<0.001	10.748	105
**miR-21**	63	42	<0.001	3.479	103
**miR-125b**	55	36	<0.001	5.664	89
**miR-200a**	65	38	<0.001	5.203	101
**miR-221**	65	41	<0.001	3.561	104
**miR-613**	65	38	<0.001	-4.268	100.119
**miR-135b**	65	41	0.005	2.645	104
**miR-136**	62	42	0.007	-2.506	101.755
**miR-200c**	65	42	0.005	2.671	70.673
**miR-519d**	65	42	0.011	2.328	105
**miR-7**	54	40	0.077	1.439	92
**miR-155**	62	39	0.376	0.316	99
**miR-181**	60	41	0.101	1.287	99
**miR-210**	59	40	0.064	1.543	73.144

	BC (n)	C (n)	p-value	t-value	df
**miR-15a+miR-16**	129	84	<0.001	11.828	211
**miR-16+miR-15a+miR-221**	195	126	<0.001	9.091	319
**miR-16+miR-15a+miR-21+miR-221**	259	168	<0.001	10.215	413.858
**miR-135b+miR-200a+miR-200c**	195	126	<0.001	6.269	228.422
**miR-15a+miR-16+miR-200a**	195	122	<0.001	4.177	216.241
**miR-15a+miR-16+miR-21+miR-125b**	260	167	<0.001	6.531	400.263
**miR-21+miR-221**	129	84	<0.001	4.993	152.08
**miR-21+miR-181a+miR-221**	187	126	<0.001	5.009	311

Table S2 n-, p-, and t-values as well as degrees of freedom (df) of independent t-tests executed on individual or multiple miRNAs

Category	Subcategory	Enrichment	P-value	P-adjusted	Q-value	Expected	Observed	miRNAs/precursors
Diseases (MNDR)	breast ductal carcinoma	over-represented	7.49E-06	3.77E-05	3.77E-05	0.156897	4	hsa-miR-15a-5p; hsa-miR-16-5p; hsa-miR-21-5p; hsa-miR-125b-5p
Diseases (MNDR)	triple-receptor negative breast cancer	over-represented	0.0022656	0.0036319	0.0036319	0.075431	2	hsa-miR-15a-5p; hsa-miR-21-5p
Diseases (MNDR)	breast cancer	over-represented	0.0023197	0.0036904	0.0036904	2.94784	7	hsa-miR-15a-5p; hsa-miR-16-5p; hsa-miR-21-5p; hsa-miR-221-5p; hsa-miR-200a-5p; hsa-miR-613; hsa-miR-125b-5p
Diseases (MNDR)	breast ductal carcinoma	over-represented	0.0032011	0.0044421	0.0044421	1.2944	5	hsa-miR-15a-5p; hsa-miR-16-5p; hsa-miR-21-5p; hsa-miR-200a-5p; hsa-miR-125b-5p
Diseases (MNDR)	female breast carcinoma	over-represented	0.0060267	0.0077963	0.0077963	0.00603448	1	hsa-miR-125b-5p
Diseases (MNDR)	breast fibroadenoma	over-represented	0.0120222	0.0145934	0.0145934	0.012069	1	hsa-miR-125b-5p
Diseases (MNDR)	estrogen-receptor positive breast cancer	over-represented	0.0120222	0.0145934	0.0145934	0.012069	1	hsa-miR-125b-5p
Diseases (MNDR)	Her2-receptor positive breast cancer	over-represented	0.0138767	0.0167477	0.0167477	1.78319	5	hsa-miR-15a-5p; hsa-miR-16-5p; hsa-miR-21-5p; hsa-miR-221-5p; hsa-miR-125b-5p
Diseases (MNDR)	breast disease	over-represented	0.0150083	0.0177396	0.0177396	0.0150862	1	hsa-miR-125b-5p
Diseases (MNDR)	breast malignant phyllodes tumor	over-represented	0.0150083	0.0177396	0.0177396	0.0150862	1	hsa-miR-21-5p
Diseases (MNDR)	progesterone-receptor negative breast cancer	over-represented	0.0175598	0.0203806	0.0203806	1.87974	5	hsa-miR-15a-5p; hsa-miR-16-5p; hsa-miR-21-5p; hsa-miR-221-5p; hsa-miR-125b-5p
Diseases (MNDR)	sporadic breast cancer	over-represented	0.0209573	0.023327	0.023327	0.0211207	1	hsa-miR-125b-5p
Diseases (MNDR)	progesterone-receptor positive breast cancer	over-represented	0.0234293	0.0259867	0.0259867	2.00647	5	hsa-miR-15a-5p; hsa-miR-16-5p; hsa-miR-21-5p; hsa-miR-221-5p; hsa-miR-125b-5p
Diseases (MNDR)	Breast Neoplasms	over-represented	0.0313749	0.0339625	0.0339625	2.14526	5	hsa-miR-15a-5p; hsa-miR-16-5p; hsa-miR-21-5p; hsa-miR-221-5p; hsa-miR-125b-5p

Table S3 miRNAs shown to be involved in breast cancer based on miRNA enrichment analysis and annotation tool (miEAA)
